# Management of Labor with Reference to Some Complications

**Published:** 1901-03

**Authors:** W. Monroe Smith

**Affiliations:** Atlanta, Ga.


### MANAGEMENT OF LABOR WITH REFERENCE TO SOME COMPLICATIONS.*

BY W. MONROE SMITH, M.D.,

ATLANTA, GA.

It is with a great deal of embarrassment that I come before you without a paper, and especially as this is the third time that I have promised to present a paper on this subject; but I was prevented from presenting one before on account of sickness, at another time on account of an obstetric case which I had to attend. Previous to this time I had a paper outlined, but I found when I came to look for it that it had been misplaced, and I have not had time to prepare another one. My remarks, therefore, will be impromptu.

It is not necessary to define labor, as every one who has had a case will readily grasp the meaning of the word. In the first place we will consider what should be carried to a confinement bed. In my case I carry a large Kelly pad, fountain syringe, a pair of obstetrical forceps, a bivalve speculum, probes, dressing forceps, absorbent cotton, iodoform gauze, hypodermic needle, bichloride of mercury and carbolic acid, etc., also the bisulphate of quinine and the fluid extract of ergot, and usually an infusion apparatus, or at least a needle which can be used on a fountain syringe as one in case of necessity.

*Remarks made before the Atlanta Society of Medicine, January 31, 1901.

In going to a case of confinement the previous history is supposed to have been already obtained, as you should have charge of the case for some time previous to the confinement. As soon as I get there I make preparations for an examination. The first examination is by palpation, that is, feeling the abdomen with the hands. Sometimes you can get points of interest in this way, and then again you can get nothing of special interest; but you can always make out a twin pregnancy if one exist. Among the things to be carried in the satchel I forgot to mention a hand brush, which, of course, should always be carried.

After palpation, I next clean my hands with soap and water, using the brush vigorously, and then clean my finger-nails carefully. I then take some antiseptic solution with some soap and sponge the genitals. I personally supervise the preparation of the confinement bed. I examine the perineum and see if there is any tear from previous labor and to what extent. I then make examination for presentation, as to whether the head, foot, or whatever part is presenting; also ascertain the amount of dilatation of the os, length of the vagina, general circumference of the pelvis, efficiency of the uterine contractions, etc.

Labor is divided into three stages, and for convenience I will take it up stage by stage. Sometimes the rupture of the bag of waters is the beginning of the first stage, but is usually defined as the time of the beginning of pains to the dilatation of the os. The time of the first stage varies from one to twelve hours, and sometimes longer. The pains should increase in severity and dilate the os, then the waters should rupture and the second stage begin. Quite frequently the bag of waters will never form, and in those cases the head has to act as a tampon to dilate the cervix, or the finger can be used for this purpose. I have had to use the finger in several cases. In using the finger be careful that you do not tear the cervix. Occasionally I find cases in which the pains come on in the first stage, and are very long or very short, and irregular and ineffectual, and the patient suffers, and yet the os does not dilate. In those cases I frequently give a dose of morphine hypodermically. The morphine usually brings about a cessation of pain, a few hours of rest and sleep, and then the pains return and labor proceeds all right. I want to emphasize this point. In such conditions some may be in favor of giving something to increase the pains, but instead of giving something to bring on the pains I give something to quiet them and give them rest, and this preserves their strength and also hastens labor materially in many cases.

After the rupturing of the waters expulsion should come on, and this stage should not last more than two or three hours. I do not allow it to last longer than this. If the pains do not come on after the head is engaged, I use something to bring them on. Of course, you should always be sure that the bladder and rectum are empty. Sometimes a hot foot-bath or a dose of whisky will bring on the pains and facilitate labor. And again when the patient is exhausted I use full doses of quinine, giving ten or fifteen grains of the bisulphate, and this gives good, strong contractions in three-quarters of an hour; if not it may be repeated. I never use the fluid extract of ergot in the second stage of labor, as I feel sure that in my early experience I had still-births as a result of this, and know other doctors who have had similar experiences. Quite recently I had a physician call me up about one o'clock one morning, and he told me that in the case which he was attending he had the cervix dilated and the child's head engaged but the pains had stopped, and he asked my advice, and I told him to give ten grains of quinine and also draw off the urine and wash out the bowels with hot water, and if that did not relieve the situation to apply forceps and deliver after waiting an hour. A few hours later he again called me up and told me that the family had called in an old physician, who had insisted upon a teaspoonful of ergot being given instead of the advice I had given; and the attending physician, being a young man, gave way to the older physician and administered the ergot, though against his scruples and better judgment. When he called me the second time he said that the woman was still in labor and asked me to come right away and help them out. I told him to watch out for the child, as I was afraid we would have a still-birth. After I reached the bedside I delivered the child in a few minutes with forceps and found it to be very much cyanosed. I began artificial respiration, but as the mother was bleeding, I turned the child over to the others while

I gave my attention to the mother. The child died in a few hours. It was well nourished and well developed, and I think its death was due to the ergot plus the prolonged sojourn in the mother's pelvis. I think it should be used only after the second stage is completed. In another instance where I had given ergot I got almost continual contraction instead of the normal intermittent contractions, and had to give chloroform and deliver with instruments to relieve my patient's distress and to save the child. Almost every time that I have used ergot I have gone away feeling that I have done a bad thing for the patient, even if I saved the child. I use quinine instead, which, if effective, gives intermittent pains, wash out the bowels, give a hot foot-bath, empty the bladder and stimulate the patient, sometimes with a hypodermic of strychnine, and these will help to bring on the pains if due to exhaustion or to simple inertia uteri.

After the child is handed over to the nurse I cleanse the perineum again, as it is more or less liable to have become infected, and cleanse my hands and introduce the finger to tell when the placenta is coming down and then kneed the uterus gently with the other hand, and in a few minutes this usually brings on firm contraction and the border of the placenta can be felt coming down and the placenta is soon delivered. In many cases the placenta is not delivered at once, the uterus being too tightly contracted to deliver, constituting hour-glass contraction, or there may be unusual firmness of the utero-placental attachment. In such cases, after waiting a reasonable time, I pass the finger up to the os, and if necessary, introduce the hand into the uterus and remove the placenta. The same way in the hour-glass contraction, if I cannot expel by Crede's method, I introduce the hand and deliver. I never feel that my patient is free from danger of hemorrhage until the uterus is thoroughly emptied, and I do not hesitate to introduce the hand into the uterus when necessary for the emptying of the uterus. If the hand is aseptic you will not infect the patient.

If you have post-partum hemorrhage, be sure the uterus is empty, inject ergot hypodermically, and if this does not control it then use the Kelly pad and wash out the uterus thoroughly with irrigating curette, using a hot carbolized solution or any sterile water. If this should not stop the hemorrhage then pack with iodoform gauze. This is something I have had to do a few times. It is not necessary to pack the vagina. I use a bivalve speculum volsella forceps to hook down the uterus, and with dressing forceps I can pack in a very few seconds. In a case recently of a primipara, the child was of full term and the first stage was more or less irregular, but by simple methods labor was brought on. The second stage was tardy but I gave quinine and delivered and there was very little hemorrhage, but enough to make some impression on the mother. I passed my hand into the uterus in this case and cleaned out and then washed the uterus out well with hot carbolated solution and the hemorrhage ceased at once, but she had become anemic and pale, so I called in another physician to assist me, as I chanced to have no means at hand with which to infuse, but my friend Dr. J. L. Campbell soon responded with infusion appliances and we gave her a subcutaneous injection of normal salt solution quite warm, elevated foot of bed, injected strychnine, ammonia, etc., and in a very short time she came around. She did not lose much blood, not as much as a great many cases that do not suffer symptoms of acute anemia, but was really threatened with death from acute anemia. She was a stout, robust woman, but very much impressed with a slight hemorrhage. I relate this case to show the great difference in the ability of women of losing blood, some quite a good deal with no symptoms and others comparatively slight amount with very serious symptoms.

This talk has been at random, but I hope I have covered most of the essentials, and I trust it will bring out a discussion.

DISCUSSION OF REMARKS MADE BY DR. MONROE SMITH.

*Dr. V. O. Hardon* (opening the discussion): I came here expecting to say something, but Dr. Smith has so completely covered the ground and gone over the subject so satisfactorily, and his views are so in accord with mine, that he has left very little for me to say, and when one has nothing to say the best thing is to say nothing. However, although I think that any man who practises obstetrics as Dr. Smith has outlined will come as near an ideal method as possible, still there are one or two points upon which we do not altogether agree, also some other points that I might touch upon to supplement his paper. I don't know that I shall take them up systematically, but rather as they shall occur to my mind.

First, in regard to ergot (and you all know my views on this); I am in full accord that it should not be given in the first or second stage of labor. I think, generally considered, that it is to be avoided at this time. As to the third stage, this should be left to the individual judgment, and mine is that it should not be given unless especially indicated. I think that when given as a regular practise in the third stage, that it is given without any positive indication for its use. Of course, there are arguments to support its use which are as obvious as in giving medicine in other conditions. But labor being a physiological process, when it is going on all right there is no reason why it should be given in the third stage. There is also a positive objection to it besides this negative one. When a woman is exhausted by a long labor, or when not a long one but rather violent, and the woman is becoming more or less exhausted by the repeated muscular contractions, to still further induce abnormal contractions by giving ergot, kept up for several hours after labor is completed, will tend still further to bring about in a woman the relaxation that favors post-partum hemorrhage, blood-clot in the uterus, and of course, the after-pains. However, I do not carry my antagonism to ergot to the extent of deprecating its use in post-partum hemorrhage. There we should use every means that can possibly be employed; and while I believe that the hemorrhage should be checked within the time that it takes the ergot to act, or the woman will be dead, yet I will not criticize any man for using it.

After the head comes down to the perineum and it does not give I frequently find it necessary to use the finger to dilate the perineum. Then comes the use of the lubricant, as it is needed there and also it is good to help the child's head through. Frequently in my practice some good old mother says: "Don't you want to use some lard?" I answer I do not, and will not allow it used in my practice. The improper selection of the lubricant will sometimes affect the labor. If I cannot get anything better I use soap, even if it irritates the baby's eyes, rather than use those things which are not sterilized. I use carbolized vaseline of two per cent. which has been boiled and put up in a special jar. I am always sure that my hands are clean and then use an antiseptic lubricant. If you use these precautions you can make as many examinations as are necessary and need have no fear of bad results from them, as I never expect to infect my patient. It has always been strange to me that some surgeons who practise obstetrics will simply wash their hands without using aseptic precautions in delivering a woman. They do not sponge off the genitals and are liable to infect their patient, and yet claim to believe in and teach antisepsis.

After the head comes down to the perineum I give chloroform freely.' In all my cases I have the woman almost anesthetized as the child's head is born, and I use more or less chloroform throughout the state. I generally deliver the child's head between contractions. I pass my fingers back in the rectum and bring the head of the child up toward the pubes. This will assist materially in saving the perineum, and the head can be easily delivered in most cases. After this I leave off the anesthetic. The constractions will come on and I deliver the shoulders. Sometimes I deliver the posterior shoulder first and sometimes the other. If the child is breathing I do not do much for it. After I get it to breathing well I get it to cry. I usually wait until the cord stops pulsating, but sometimes this will continue indefinitely, and in those cases I wait a reasonable length of time before clipping it. The reason for this is that the child whose cord has not been cut soon will be stouter and stronger than one who has been treated otherwise, other things being equal.

Ancther thing is the use of forceps. Where I do not have contractions sufficient to deliver the child and the head has remained in one position for an hour or thereabout and there seems to be no strengthening of the pains, I do not hesitate to deliver with the forceps. I not only consider it safe but safer than the waiting. Only yesterday morning a doctor had been with the patient all night, and in the second stage for hours, and he sent for me and I found a good large succedaneum forming on the child's head, which was on the perineum and not delivered. I delivered in a few minutes without forceps, but would have used forceps if it had not been delivered immediately without them. The child was very much cyanosed and this could have been prevented by the use of forceps at an earlier hour, and the application of forceps is a safe operation with asepsis. I have done the operation quite a number of times and am still more and more ready to apply them.

After the delivery of the child comes the third stage of labor. After the second stage is completed I frequently give a teaspoonful of ergot. The statement is made that ergot given after the child is born will, in some cases, produce hour-glass contraction. I do not think it will do this, as we expect to get the effect of the ergot not sooner than thirty or forty minutes after its administration, and certainly we should have the uterus emptied by the expiration of that time. If premature retraction of uterus should occur, it is readily and easily managed. I like to feel that I have given something that will bring about a good firm retraction of the uterus and prevent the possibility of post-partum hemorrhage from lack of tonicity, which is likely to occur in most cases.

As to the dilatation of the cervix: Dr. Smith said that in the first stage as it progresses the bag of waters coming down produces dilatation of the cervix. If we relied entirely upon this hydrostatic pressure the cervix would rarely dilate. It is here that another physiological function comes into play, and that is, the antagonism between the muscular structures of the uterus and the cervix; when one contracts the other dilates. This antagonism is proven by cases of transverse presentation.

There was one point in regard to the management of the perineum which was not mentioned by Dr. Smith. If the vertex be presenting and the occiput to the front (as it is in 95 per cent. or more of cases), the vertex will be under the pubic arch and that is the time that the strain comes on the perineum. Of course, we know the different diameters of the head, and the safety of the perineum depends upon the short diameters coming into the long diameters of the vulva, and where there is complete flexion then this happens, but if extension takes place then the reverse will be true. So I deem it of the greatest importance that we should not allow extension to take place until the pubic arch covers the back of the neck of the child. This can be demonstrated at any time by experiment. In my practice I consider this the most important means of saving the perineum.

Another point not touched upon which I consider of some value. When the head comes down and presses on the perineum, sometimes there is a pause in the labor. Sometimes it will stop right here and hold up for a considerable time. This is rather dangerous for the child if delayed very long. Long labors produce cyanosis, as Dr. Smith has said. Now, if we want to excite uterine contractions, I know of no better way than to hook the finger in the posterior commissure and press downward toward the anus. This will always excite contractions as surely as anything I know of. I always make use of this at this point.

Dr. Smith says he waits until the pulsation of the cord ceases before he cuts it, in order to give the child as much blood as possible. Well, I never could see any advantage in that. It does not make any difference how long you wait there is no suction by means of which the blood is returned to the heart of the child. If you wait until the pulsation stops and then cut the cord you will find that the placenta is still filled with blood. I have never seen any difference in the child, but it may be an error of observation on my part. It is a minor point, and I simply present this as my opinion.

Mr. President, in the management of labor there are certain general principles, and if we bear them in mind we will not go far wrong. Labor is a physiological process. No one deprecates "meddlesome midwifery" more than I do. In perfect cases nothing is to be done but to simply sit by and see that nothing goes wrong. But it is impossible to know this unless you investigate, and this cannot be done without a vaginal examination, but, and here I speak, or ought to speak cautiously, I believe the majority of men (and I say this advisedly) do not make the most of the opportunity to see the condition which exists. I believe that the majority of men do not make a diagnosis of the position of the child. They know that the head is presenting but not what part. There is a normal and an abnormal presentation of the head, and it is in the abnormal that something should be done. It does not require an expert to make out the position and presentation. The data are there for diagnosis and they are as plain to a man of limited experience as otherwise, and I believe that some complicated labors which result in the death of the child and exhaustion of the mother are due to a failure to make a diagnosis of the presentation and act accordingly. I saw a case a few years ago in which labor had been going on for three days, and I was called in by the physician and found a brow presentation which had not been recognized. This was a condition for forceps, as labor is so long as to cause the death of the child. After delivery with forceps the child could not be revived as it was dead. This was the only child which the woman ever had, and she would have given anything for one, and this child was sacrificed simply through failure to make an early diagnosis. I have seen such cases often, and it was because the men did not avail themselves of all the data in order to make an early diagnosis. However, there are times when even the most expert cannot make a diagnosis, as when the caput succedaneum has formed, but even then it can be done by palpation.

Another cardinal principle to which Dr. Smith has alluded, is the necessity of asepsis. Here again there are many lapses in the practice of obstetrics. As he has said, complete asepsis without a break in the chain is as essential as in any operation. You have the same conditions. You have an open wound and the dangers of absorption of septic matter, and it should be treated as a wound at the bottom of a sinus. Just as a wound must be sterile, and the sinus which leads to it, just so in labor the uterus must be sterile during the process and several days following it. This can only be accomplished by absolute sterilization of everything which comes in contact with it. In fact, there is greater danger than in a surgical operation, and I would have less dread of infection by having my abdomen opened and an unsterilized finger thrust into it than into a woman's uterus after labor; for the uterus is in a condition of absorption, which begins to take place immediately, and is eager to take up everything. The abdomen attempts to get rid of it through the emunctories, but from the womb it is carried to all parts of the system.

The first thing is to have complete sterilization of the passage, as it is normally so, and anything carried in is on the hands of the physician. As in a wound, the second thing is to get rid of any foreign matter; and the third is to get complete drainage, as after an operation. This last is usually a point which is overlooked. Usually the woman lies in bed without being raised up at all, and a pocket is formed at the upper part of the vagina and this collects all matter. For several years I have made it a rule that the patient shall be raised into an upright position at least twice a day, and especially when emptying the bowels, and in this way everything is drained by the force of gravitation; and also, when they sit up in this position, clots which would not otherwise escape, pass out. Have complete sterilization, and complete removal of all foreign matter, and thorough drainage, and by such treatment you will have few cases of sepsis.

As to instrumental intrusions. I believe with Dr. Smith, that the proper use of forceps is a perfectly safe operation. I have never seen any harm come from the use of them, but I have seen harm result from a failure to use them. I have seen cases in which the child's life was lost simply because forceps was not used. If a woman was worn out, and even though everything was normal, but progressing slowly, I would not hesitate to save that woman from twelve hours of pain by delivering with forceps, no more than I would hesitate to remove the placenta from the womb, either by Crede's method, or by hand. It is simply to relieve her of an unpleasant condition, and I would use forceps under those circumstances, although this is not in accord with the generally accepted views. I believe that more women suffer than is neccessary, for they could be delivered by forceps sooner than they are. Of course, there are cases where the use of forceps is imperative, but in these cases it is optional. I do not believe in meddlesome midwifery, but where the procedure is as safe as this and will save many hours of suffering and exhaustion, I see no reason why forceps should not be resorted to.

*Dr. Baird:* I would like to inquire of Dr. Hardon as to whether he gives an anesthetic in the use of forceps?

*Dr. Hardon*: I invariably use an anesthetic.

*Dr. Baird:* I recall that some years ago the Society devoted one evening to the discussion of normal labor, and it was quite interesting to hear the different views, in fact, it was the most interesting, entertaining and instructive of any meeting of the Society.

As to the use of ergot, I generally find it best to wait until the uterus is empty before giving it. It is administered to avoid any trouble in the removal of the placenta, and I feel more comfortable myself to give ergot, and generally do for my own peace of mind. While I have never had any great trouble from hemorrhage, I have always dreaded it and appreciate the dangers and difficulties from it and, therefore, avoid it as much as possible. While labor is a physiological process, yet the subject is not always in a physiological condition, and therefore, we cannot always expect normal results; and we are, therefore, justified in rendering assistance for want of physiological procedures. I do not think the ergot exhausts the woman in any way. There must always be a certain amount of contraction of the muscular tissue of the uterus, and where it is lacking the ergot supplies this. I think it lessens the liability to after-pains by making a firm contraction in place of the alternating contractions which may result. It is on the safe side, and I feel much more comfortable in my own mind, and if there is an accident afterward I would feel that this much had been accomplished in advance.

A very important point is to watch the uterus for one hour after delivery. I prefer to have the baby turned over to a nurse while I see that the uterus is firmly contracted. I have a dry towel placed under the hips and do not remove the clothes until after the baby is dressed, and in the meantime I sit with my hand over the fundus to see that there is a firm contraction. It usually takes the nurse about one hour to dress the child, and if at the end of that time I am not sure of the contraction of the uterus, I give the nurse something else to look after, and thus delay things until the contraction takes place. I sometimes stay as long as two hours after the baby is born, and the first hour is spent with my hand on the uterus. I believe that most men attend to the baby and have the woman fixed at once, but I reverse the procedure.

*Dr. J. L. Campbell:* I have nothing to add to what has been said, but I have enjoyed the paper very much, and agree with Dr.

Smith in full. I have not used ergot, and therefore cannot say anything about it, but I have enjoyed the discussion very much.

*Dr. Duncan:* I enjoyed the paper very much, and agree in the main with everything that Dr. Smith said. As to ergot, I formerly used it and never went to a case of labor without it. But I have had no occasion to carry it with me in the last twenty years, and I never used it in the first or second stage. Some twenty years ago I used it after the second stage, but since then I have not used it at all.

As regards washing out of the uterus after delivery of the placenta in case of instrumental labor and attached placenta, I prefer using sterilized water. I do not use any carbolic acid or salt with it. Where there is a tendency to great depression I give the patient drinks of hot water, as hot as can be taken, and use hot normal salt solution by the rectum. I get good results by using these in large quantities.

I think that asepsis is a good thing in obstetrics and follow out the plan suggested by Dr. Smith, but I have seen many cases where the lard was used as an unguent without any bad results, and I think that a good article of lard will answer where you cannot get anything else.

*Dr. Hubbard:* There is a point about which I can speak very feelingly from one experience, and that is post-partum hemorrhage. I do not believe in large doses of ergot in the first, second, or even the third stage of labor, as I never give it in large doses. I was so unfortunate one time as to have a case of post-partum hemorrhage, and as Dr. Hardon very well said, I do not think ergot could have had time to do any good. I immediately went into the uterus and cleared it out and used the other hand over the uterus to get up contractions, and gave ergot, but I do not think it does any good where you already have the hemorrhage. As a preventive it is good but not in teaspoonful doses. In ten or fifteen drop doses it is a good thing and will do no harm. You have to watch the dose of medicines as well as the effect of the drug, and this is the case with ergot. Medium doses in the third stage, as Dr. Baird says, will leave a better feeling in the mind.

In the use of forceps, I think the sins against the forceps are not the number of times they have been used, but the way they have been used. If they are used properly, actually no danger can occur, and it will save life in a great many instances. It has been my fortune to use forceps in more cases than to have normal labors. I do not wait too long before using the forceps, and I am thoroughly in accord with the idea of using instruments and using them properly.

I think that Dr. Smith has covered the ground thoroughly, and it could not have been a better paper if he had written it out.

*Dr. Baird:* In the use of lubricants, I have never found it necessary to use anything for the finger, as I have found material supplied by the vagina all that is necessary to use. I simply cleans and sterilize the hands.

Dr. Duncan spoke of washing out the uterus. I never use any injection of any sort, and I never have any douches unless there is positive indication for their use. A few years ago it was very fashionable to use the douche, but so far as I know, it is now out of style. I think it was favored by Dr. Thomas of New York, but is now out of practice. I remember writing an article on this subject several years ago in which I took the ground against the use of it.

*Dr. Duncan:* My reference to washing out the uterus was only in cases where you wanted to arrest hemorrhage and not in normal labor.

*Dr. Hardon:* As regards the use of lubricants, my experience has been the same as Dr. Baird's, and I have never found it necessary to use anything. I think that where a man attempts to pull a child out by the foot he will find it pretty well lubricated. I wash my hands in bichloride and I have found even this does not interfere with the lubricant.

One point Dr. Baird mentioned which I intended to speak of, and that was the watching of the womb to see that the contractions which follow the expulsion of the placenta are kept up. Some years ago Dr. Baird spoke of this point. It was a new idea to to me and impressed me at the time, and since then I have carried it out in all my cases of labor. I have always deemed it unwise to wash and clean up the woman before she has had rest. I simply wait for the dressing of the child and sit and talk with the friends. Since Dr. Baird mentioned this I have carried it out and taught it at the college.

In regard to washing out the uterus it was, as Dr. Duncan said, in cases of hemorrhage. As Dr. Baird mentioned, it was the fashion to use douches after normal labor when antiseptics first came in, but now they are never used.

*Dr. Smith* (closing the discussion): I have been very much gratified and also very grateful for the discussion.

In the use of lubricants my experience differs from the gentlemen who have just been speaking. It is very seldom that we need a lubricant to make an examination, but after the child's head comes down we have a dry, almost astringent condition of the mucous membrane, and in these cases I use the lubricant freely, and surely with benefit to my patient.

One point which I forgot to mention was the attention to be given to the perineum after the birth of the child. I always examine the perineum, and if I find a tear sufficient to warrant even one stitch, I take that stitch then. During my work I have found several women with torn perineums which prominent physicians would not repair at the time because of the exhaustion of the patient. I will not only repair the perineum after exhausted condition, but have done it even after the post-partum hemorrhage. I have used catgut a few times, but after two weeks you will find little places which have not united. Also, have used silk a few times, but on account of abscess do not use this any more. I have adopted silver wire and always carry this with me. I leave it in for ten or twelve days and then remove it, and in almost every case find I have perfect union.

In regard to having the patient dressed early, I differ very materially from the gentlemen who have spoken. It is my custom always to have an attendant to place the hand over the uterus and instruct them to hold the hand firmly, and if she finds the knot is disappearing to let me know. While she is doing that I sponge the thighs and genitals and have the husband to slip out the things which have been placed over the sheet to catch the discharges, and then put on dry clothes. I examine the perineum, and if torn I

repair this next, and then I take some gauze or absorbent cotton, or soft cotton cloth and fold it ready to use and apply it to the genitals. After this I examine the uterus and be sure that it is firmly contracted. I rarely watch it longer than thirty or forty minutes. It usually requires about one hour to get through with the patient, and after that I never remain longer to watch the uterus.

*Dr. Hardon:* I want to ask one question for information. I formerly used silver wire to repair the perineum, but found that in renewing the dressings that the application of the pad gave pain on account of contact with the ends of the wire, and also that things could not be kept clean with the wire there because of the pain while washing the parts and the wire caused a great deal of irritation, and I would ask how the doctor obviates this?

*Dr. Baird:* I would like to ask another question. The immediate repair of the perineum is recognized, and for a number of years I used the silver wire, but for the past six or eight years have used nothing but silk, but not for the reason spoken of by Dr. Hardon, but because the wire causes more pain in making the stitches than the passage of the needle. I used the curved needle, and the wire was threaded by means of intervening silk thread. After the needle passes, the silk follows, and then the wire has to be dragged through the tissues, and this caused so much trouble that I gave it up.

*Dr. Campbell:* I would like to mention that for the repair of the perineum I have used the silkworm gut with the best results of all. I get the same result with silk as mentioned by Dr. Smith. I never saw better results with anything else than with the silkworm gut.

*Dr. Smith:* I am very glad that Dr. Baird and Dr. Hardon brought out those points. I feel that I can help Dr. Baird. I carry a number of curved needles, for some will break. I use a good sized needle which will reach from one side to the other, and do not use any silk, but put the wire directly into the needle. I have a sharp-pointed needle and grasp it with the needle-holder, and pass the finger into the rectum to direct the needle, and after passing the needle there is practically no trouble or pain from the wire. I

never have any abscess following the use of the silver wire. I have seen a little trouble from the mechanical irritation of the wire, but this is very slight. The pads which I use for dressings are never made of absorbent cotton, and I always instruct the nurse to use a carbolic solution and wash the perineum by sight, and make little mops and sponge around the stitches and thus avoid mechanical irritation.

